# Supplementary material for: 3D virtual histopathology of cardiac tissue from Covid-19 patients based on phase-contrast X-ray tomography
Source: eLife. 2021 Dec 21;10:e71359. doi: 10.7554/eLife.71359 (PMC8747495; doi:10.7554/eLife.71359)
Supplement: Transparent reporting form [file elife-71359-transrepform1.docx]

***eLife’s* transparent reporting form**

We encourage authors to provide detailed information *within their submission* to facilitate the interpretation and replication of experiments. Authors can upload supporting documentation to indicate the use of appropriate reporting guidelines for health-related research (see [EQUATOR Network](http://www.equator-network.org/  \t _blank)), life science research (see the [BioSharing Information Resource](https://biosharing.org/)), or the [ARRIVE guidelines](http://www.plosbiology.org/article/info:doi/10.1371/journal.pbio.1000412) for reporting work involving animal research. Where applicable, authors should refer to any relevant reporting standards documents in this form.

If you have any questions, please consult our Journal Policies and/or contact us: [editorial@elifesciences.org](mailto:editorial@elifesciences.org).

**Sample-size estimation**

- You should state whether an appropriate sample size was computed when the study was being designed
- You should state the statistical method of sample size computation and any required assumptions
- If no explicit power analysis was used, you should describe how you decided what sample (replicate) size (number) to use

Please outline where this information can be found within the submission (e.g., sections or figure legends), or explain why this information doesn’t apply to your submission:

**Replicates**

- You should report how often each experiment was performed

In the Methods & Materials Section (Tab.1) we give the size of each sample group, further information about clinical background as well as the results of the structure tensor analysis is given in the Appendix Tab.1 and 2;

Since the work is about a new technique for the quantification of cardiac tissue, it presents illustrative cases. For the quantification of the vascular network, one control sample was compared to a Covid-19 sample. This method is intended to show the increased level of perforated capillaries in Covid-19. The results are supported by high resolution reconstructions and SEM recordings.

- You should include a definition of biological versus technical replication
- The data obtained should be provided and sufficient information should be provided to indicate the number of independent biological and/or technical replicates
- If you encountered any outliers, you should describe how these were handled
- Criteria for exclusion/inclusion of data should be clearly stated
- High-throughput sequence data should be uploaded before submission, with a private link for reviewers provided (these are available from both GEO and ArrayExpress)

Please outline where this information can be found within the submission (e.g., sections or figure legends), or explain why this information doesn’t apply to your submission:

**Statistical reporting**

Relevant here are technical replicates in the tomography, which are provided by observation of the same structure in different magnification and at different photon energy. Also relevant is the phase retrieval process. This information is also described in the M&M section.

- Statistical analysis methods should be described and justified
- Raw data should be presented in figures whenever informative to do so (typically when N per group is less than 10)
- For each experiment, you should identify the statistical tests used, exact values of N, definitions of center, methods of multiple test correction, and dispersion and precision measures (e.g., mean, median, SD, SEM, confidence intervals; and, for the major substantive results, a measure of effect size (e.g., Pearson's r, Cohen's d)
- Report exact p-values wherever possible alongside the summary statistics and 95% confidence intervals. These should be reported for all key questions and not only when the p-value is less than 0.05.

Please outline where this information can be found within the submission (e.g., sections or figure legends), or explain why this information doesn’t apply to your submission:

(For large datasets, or papers with a very large number of statistical tests, you may upload a single table file with tests, Ns, etc., with reference to sections in the manuscript.)

For each sample the parameters obtained from the structure tensor analysis of the laboratory data is given in Appendix Tab.2. The results of the statistical analysis are also plotted in Fig.5. This section is intended to describe a new way of analyzing data; no special hypothesis was tested.

**Group allocation**

- Indicate how samples were allocated into experimental groups (in the case of clinical studies, please specify allocation to treatment method); if randomization was used, please also state if restricted randomization was applied
- Indicate if masking was used during group allocation, data collection and/or data analysis

Please outline where this information can be found within the submission (e.g., sections or figure legends), or explain why this information doesn’t apply to your submission:

**Additional data files (“source data”)**

The samples were grouped based on the cause of death, confirmed by pathological examinations.

- We encourage you to upload relevant additional data files, such as numerical data that are represented as a graph in a figure, or as a summary table
- Where provided, these should be in the most useful format, and they can be uploaded as “Source data” files linked to a main figure or table
- Include model definition files including the full list of parameters used
- Include code used for data analysis (e.g., R, MatLab)
- Avoid stating that data files are “available upon request”

Please indicate the figures or tables for which source data files have been provided:

Microscopy images of HE stained sections of all samples are shown in Appendix Fig. 1.

The results of the structure tensor analysis of the laboratory is given in Appendix Tab.2; synchrotron results are shown in Appendix Fig.3. The tomographic datasets recorded in WG conﬁguration as well as the PB datasets used for the segmentation of the vascular system were uploaded to Zenodo (see link in MS). Additional data (raw data, PB and laboratory reconstructions, structure tensor analysis) is curated here at University of Göttingen and at DESY can be obtained upon request from the authors; due to the extremely large size >15TB it cannot presently be uploaded easily to a public repository. The code used for segmentation is provided at github.com/patmjen/blood-vessel-segmentation.
